# Prospective assessment of stress and health concerns of radiation oncology staff during the COVID-19 pandemic

**DOI:** 10.1016/j.ctro.2022.06.001

**Published:** 2022-06-08

**Authors:** Sebastian M. Christ, Michael Denner, Nicolaus Andratschke, Panagiotis Balermpas, Brigitte Hilty, Stephanie Tanadini-Lang, Lotte Wilke, Sophie Perryck, Matthias Guckenberger

**Affiliations:** Department of Radiation Oncology, University Hospital Zurich, University of Zurich, Zurich, Switzerland

**Keywords:** COVID-19, Corona virus disease of 2019, HR, Human resources, PPE, Personal protective equipment, RTT, Radiation therapy technician, SD, Standard deviation, SOP, Standard operating procedure, USZ, University Hospital Zurich, UZH, University of Zurich, WHO, World Health Organization, COVID-19, Radiation oncology, Healthcare worker stress

## Abstract

**Introduction and background:**

The COVID-19 pandemic has required rapid and repetitive adjustment of radiotherapy practice, hospital-level and department-level organization and hygiene measures. To prospectively monitor and manage stress levels and health concerns, employees of a radiation oncology department were invited to participate in weekly online surveys during the first year of the pandemic.

**Materials and methods:**

Starting March 31st, 2020, cross-sectional online surveys were distributed to all employees of the Department of Radiation Oncology, University Hospital Zurich. The survey included questions about the profession, the work setting, the global stress level as well as the health concerns during the past work week. Stress levels and health concerns were assessed on a 10-point scale. SurveyMonkey® was used to conduct the survey. Distribution was performed via email. Participation was anonymous and voluntary.

**Results:**

Between March 2020 and February 2021, 50 weekly surveys were distributed to 127 employees on average and resulted in 1,877 individual responses. The average response rate was 30%. The mean global stress level varied significantly by profession, ranging from 2.7 (±2.5) points for administrative staff to 6.9 (±2.3) points for radiation therapy technicians (p < 0.001). The mean global stress level was highest with 4.8 (±2.9) points for in-hospital work with direct patient contact. Health concerns were highest regarding family and friends with 4.0 (±3.1) points on average. Changes of the stress level varied in correlation with infection waves.

**Conclusion:**

Weekly online surveys for prospective assessment of stress levels and health concerns were successfully conducted during the first year of the COVID-19 pandemic, indicating their feasibility and value to monitor profession and workplace specific stress patterns and to allowed for tailored interventions. The physical and mental health of frontline healthcare workers in radiation oncology should remain a top priority for departmental leadership beyond the COVID-19 pandemic.

## Highlights

• The COVID-19 pandemic required continuous adjustment of radiotherapy practice.
• Global stress levels and health concerns of staff followed COVID-19 infection waves.
• Frontline workers with direct patient contact were most affected by the pandemic.
• Beyond COVID-19, weekly regular online surveys can help to monitor staff well-being.

## Introduction and background

On March 11, 2020, COVID-19 was declared a pandemic by the World Health Organization (WHO) [1]. Ever since, COVID-19 has put healthcare systems all around the world under enormous pressure and caused them to operate in a constant state of emergency. From March 2020 to March 2021, i.e., during the time including the first and second COVID-19 waves, there were 604,020 registered positive COVID-19 cases in Switzerland, highlighting the substantial case load in the country [2]. From the start of the COVID-19 pandemic, massive and continuous changes to hospital operations were therefore implemented across Switzerland. Major radiation oncology centers mostly continued oncological treatment at pre-pandemic levels, which in turn required rapid and repetitive adjustments of radiotherapy routines, hospital-level and departmental-level processes as well as hygiene measures [3]. All radiation oncology members of staff had to accept a crucial role to achieve this continuation of cancer care in this time of serious uncertainties. It is well documented in the literature that routine work during the extreme conditions of a pandemic can have detrimental health effects on healthcare staff, which also applies to the recent COVID-19 pandemic [4], [5], [6].

So far, there have been very few reports examining stress level and mental health problems in radiation oncology departments during the COVID-19 pandemic. Those studies focused on sub-groups of departmental staff such as radiation therapy technicians (RTTs) in Canada and Norway [7], or radiation oncology residents in Italy [8]. To our knowledge, no study has assessed comprehensively stress levels and health concerns across all professions in a single radiation oncology department and no study has continuously assessed stress levels for a long duration of the first year of the COVID-19 pandemic. In this study, we report and analyze prospectively stress levels and health concerns of all employees at an academic radiation oncology department. We also qualitatively describe measures, which have been implemented with the aim to curb infections as well as to decrease psychological distress of healthcare workers during the first year of the COVID-19 pandemic.

## Materials and methods

### Study population

All employees of the Department of Radiation Oncology of the University Hospital Zurich (USZ) with an active contract between March 2020 and February 2021 were eligible for participation in this study. Professions surveyed included administrative staff, clinicians, medical physicists, nurses, nursing assistants, research staff, RTTs, and “other” professions. Participation in the study was voluntary, anonymous and unpaid. Completion of the questionnaire took place during working hours.

### Survey instrument

Starting from March 31st, 2020, a survey was sent out to all department staff via their professional email address on a weekly basis. The email always included a link to the survey, an explanation about the context of the study, a reminder that participation in the survey was voluntary and anonymous, yet that participation was encouraged with the aim to improve employee well-being and satisfaction. The survey was designed by senior staff from different professions working in our department. The design of the survey was informed by other routine surveys conducted by the human resources (HR) department at the USZ as well as by a thorough review of the literature [9], [10], [11], [12]. The survey consisted of six questions and was conducted in English, as our department is very international with English as working language in several contexts. After consenting to participate, respondents were first asked to select their profession from a drop-down menu. Secondly, it was specified whether work during the last week had been mostly performed onsite in the hospital, with or without patient contact, or from remote in the home office setting. In addition, respondents specified on a 10-point scale: (1) global stress level during the past week (“0″ = no stress; “10” = maximal stress), (2) concerns about their own health during the past week (“0” = no concern; “10” = maximal concern), (3) concern about the health of family and friends during the past week (“0” = no concern; “10” = maximal concern), and (4) concerns about their patients’ health during the past week (“0” = no concern; “10” = maximal concern). A returned survey was counted as one completed survey response, even if a piece of information was missing. The online survey solution SurveyMonkey® was used to conduct the survey (Supplement 1).

### Data analysis

Descriptive statistics such as mean and standard deviation (SD) were calculated for all variables under study. The Kruskal-Wallis test was employed to assess statistically significant differences between groups. Statistical significance was set at p = 0.05. All statistical analyses were carried out using the software package STATA® (v.16.1). For visualization purposes, appropriate figures were created using the software Microsoft® Power BI Desktop® (v.2.9).

## Results

### Study population

Between March 31st, 2020 and February 17th, 2021, an average of n = 127 employees worked at our Department of Radiation Oncology. There were n = 28 (22%) RTTs, n = 22 (17%) clinicians, n = 22 (17%) research staff, n = 18 (14%) administrative staff, n = 15 (12%) medical physicists, n = 10 (8%) nurses, n = 7 (6%) nursing assistants, and n = 5 (4%) employees with other professions.

### Survey participation rates

Across the whole year starting March, 31st, 2020, a total of 50 online surveys were distributed, resulting in 1,877 responses. The overall average response rate across all professions was about 30%. Average response rates differed by profession and time point. Medical physicists had an average rate of 62%, while research staff and nursing assistants had average rates of 20% and 5%, respectively (Fig. 1a). The highest and lowest weekly response rates were 43% and 20%, respectively (Fig. 1b).

**Fig. 1a:**
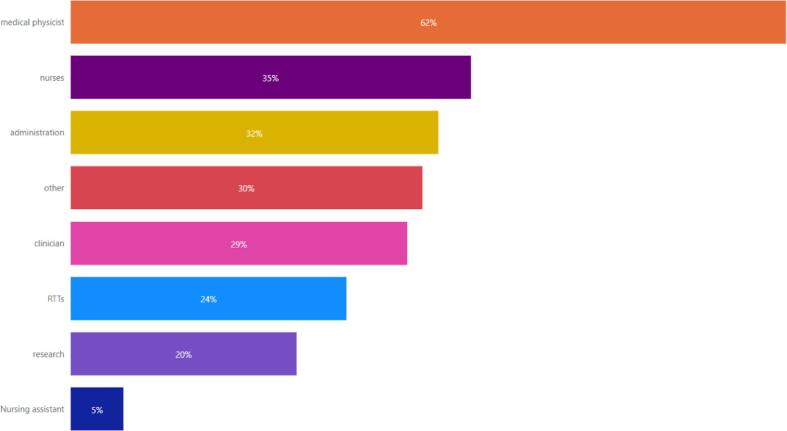
Average response rate by profession*.

**Fig. 1b:**
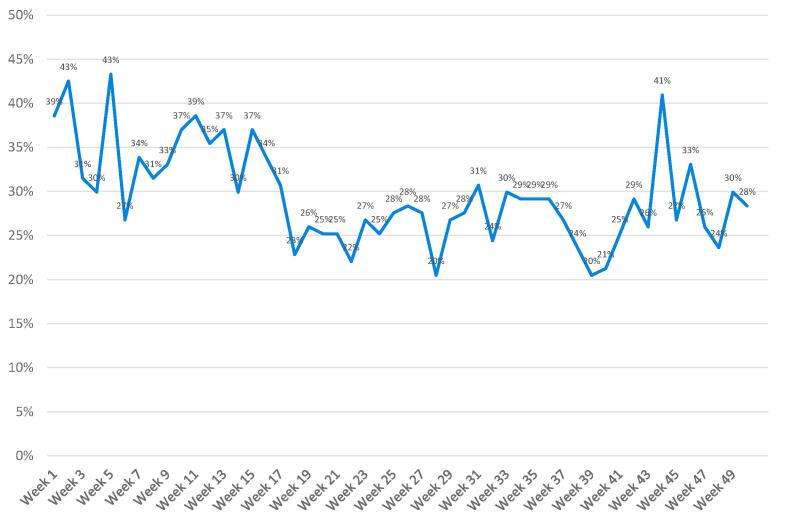
Response rates over time*.

### Survey responses on stress levels and health concerns

The average global stress level varied significantly between professions (p < 0.001). RTTs had the highest average global stress level with 6.9 (SD, 2.3) points, followed by nursing assistants and medical physicists with 4.4 (SD, 2.8) and 4.2 (SD, 2.8) points, respectively. The lowest average global stress level was reported by research and administrative staff, with rates of 2.9 (SD, 2.1) and 2.7 (2.5) points, respectively. When compared to all other professions, RTTs had the highest combination of point scores of global stress level and health concerns (p < 0.001). With respect to health concerns, all employees were most concerned about the health of family and friends, with an average of 4.0 (SD, 3.1) points (p < 0.001). Concerns about the health of patients came second, with an average of 3.6 (SD, 3.1) points, while the concern about one’s own health was scored lowest with 3.1 (SD, 3.0) points on average. Sub-group analysis with respect to profession showed statistically significant differences (p < 0.001): RTTs had the greatest health concerns, followed by medical physicists and lowest values were observed for administrative and other staff. While RTTs were, on average, similarly concerned about the health of family and friends as well as patients with 7.2 (SD, 2.3) points, they were, on average, less concerned about their own health (6.1; SD, 2.9 points). For all other professions, the concerns about health were strongest about family and friends and lower about their own health and the health of patients. An overview of health concerns by profession is given in Fig. 2a and Fig. 2b. Fig. 3a, Fig. 3b and Fig. 3c illustrate that both global stress level and health concerns by sub-category were almost twice as high for RTTs than for other professions such as nursing assistants, medical physicists and nurses. Despite no baseline data for global stress level and health concerns being available for our survey, an almost two-fold inter-professional difference was interpreted as very high or “toxic” stress levels, requiring special attention from departmental leadership.

**Fig. 2a:**
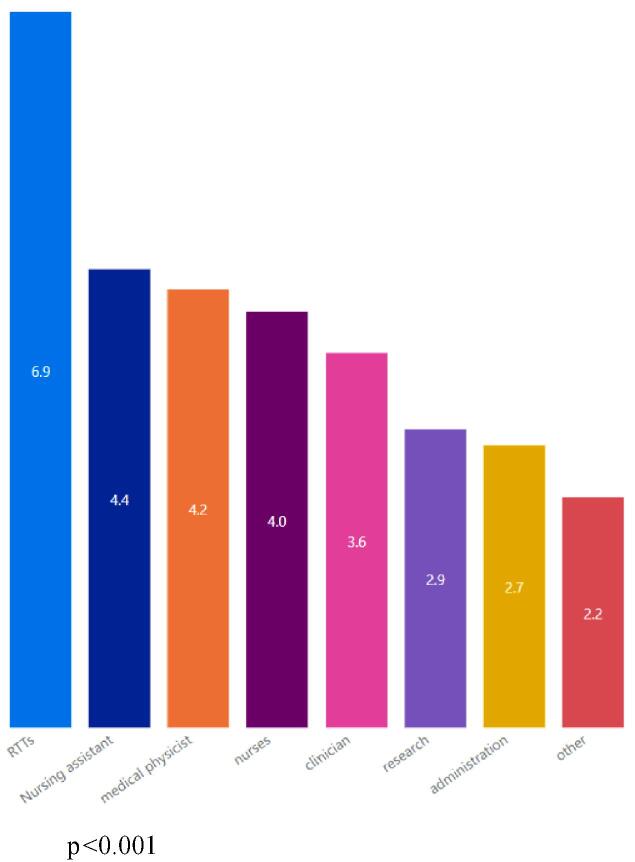
Average global stress level (10-point scale) by profession.

**Fig. 2b:**
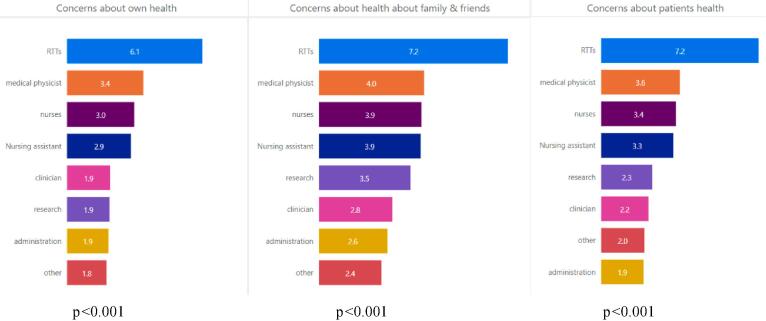
Average health concerns by sub-category (10-point scale) and by profession.

**Fig. 3a:**
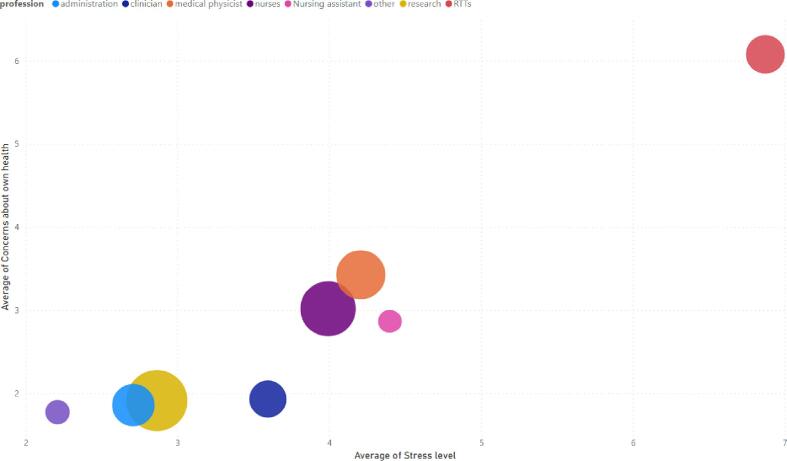
Average global stress level and own health concern (10-point scale).

**Fig. 3b:**
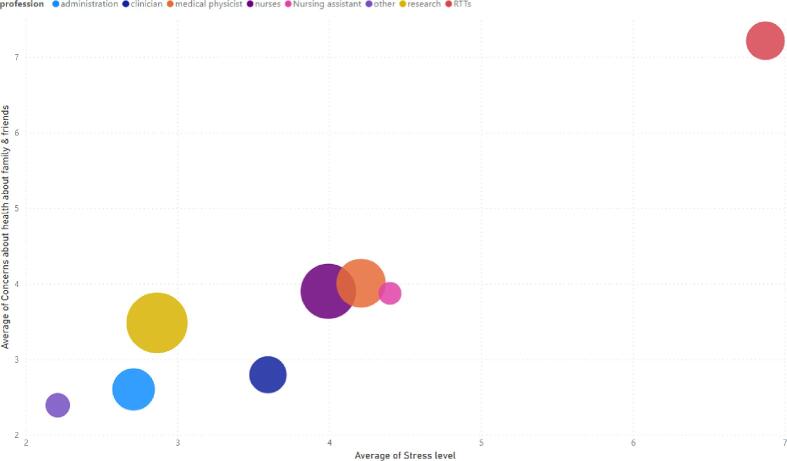
Average global stress level and health concern for loved ones (10-point scale).

**Fig. 3c:**
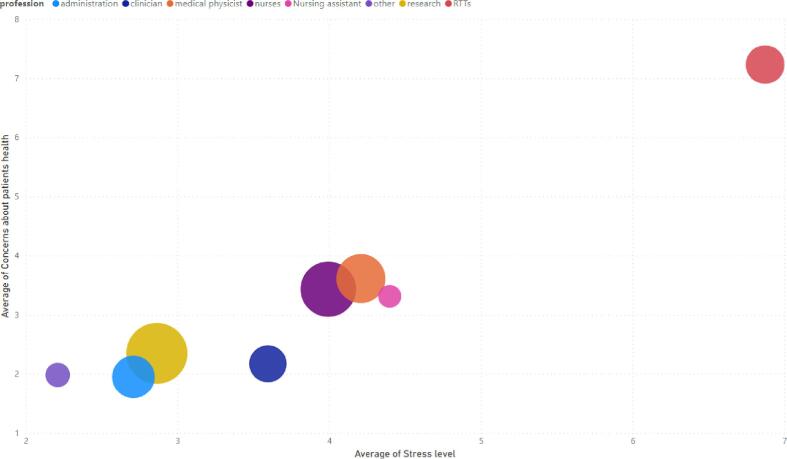
Average global stress level and health concern for patients (10-point scale).

### Responses by work locations

Across all professions, the global stress level was highest for in-hospital work with patient contact with an average of 4.8 (SD, 2.9) points, whereas the global stress level was slightly, but significantly different for in-hospital work without patient contact and home-office with 3.5 (SD, 2.8) and 3.7 (2.6) points, respectively (p < 0.001). The same pattern was observed for the sub-categories of health concerns per work location, with differences also being highly statistically significant (p < 0.001). Health concerns were generally highest for the group of employees conducting in-hospital work with patient contact. In this work location, concerns about the health of family and friends were scored with 4.4 (SD, 3.3) points on average, concerns about the health of patients and one’s own health with 4.2 (SD, 3.3) and 3.6 (SD, 3.1) points, respectively. For a detailed overview of global stress level and health concerns by work location, consult Fig. 4a and Fig. 4b.

**Fig. 4a:**
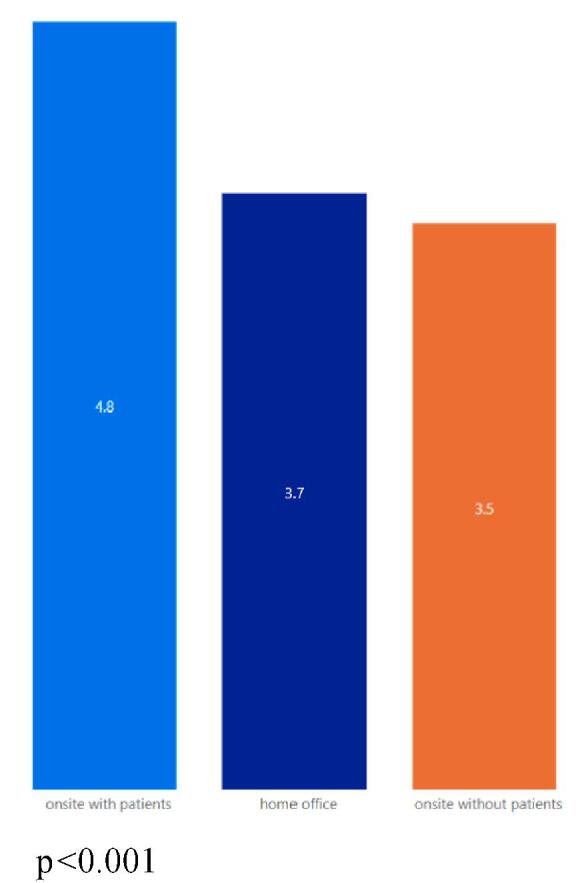
Average global stress level (10-point scale) by work location.

**Fig. 4b:**
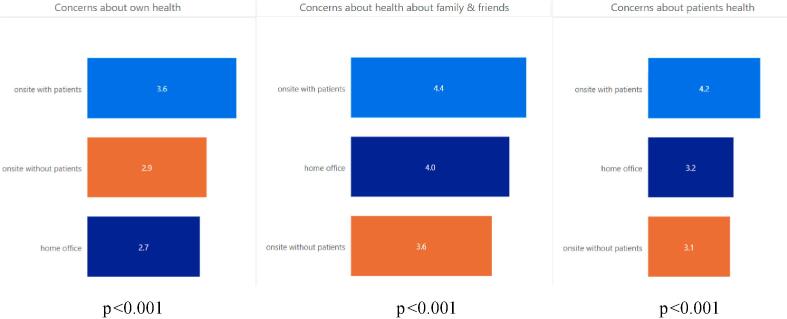
Average health concerns by sub-category (10-point scale) and by work location.

### Time trends and other patterns

When analyzing the time trends of the global stress level and health concerns during the first year of the pandemic, average global stress level was 4.2 (SD, 2.8) points in March/April 2020. It was lower during the summer months of 2020 and peaked again in November and December 2020 with an average of 4.7 (SD, 2.1) and 4.8 (SD, 2.9) points, respectively, before falling off to 4.3 (SD, 2.0) points by March 2021. The health concern curves followed a similar pattern. While the average point score for concern about health of family and friends was higher than the global stress level in March/April 2020 (4.9; SD 2.9 points), it fell below the global stress level in November and December 2020. These time trends are illustrated in Fig. 5a and the time course of COVID-19 cases, hospitalizations and deaths from March 2020 until March 2021 in Switzerland is illustrated in Fig. 5b. The regression analysis resulted in a correlation coefficient is 0.097 between the global stress levels and daily new COVID-19 cases (Fig. 5c).

**Fig. 5a:**
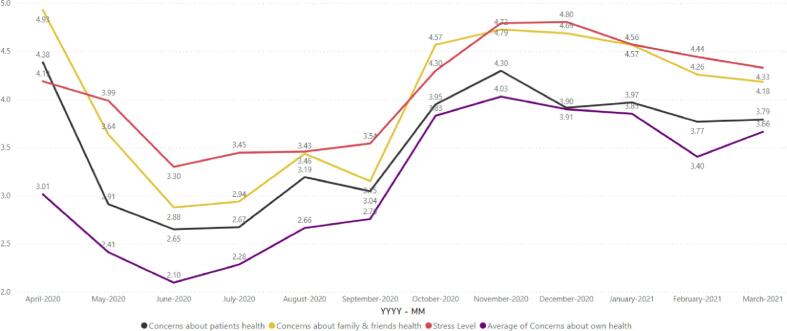
Average global stress level and health concerns (10-point scale) over time.

**Fig. 5b:**
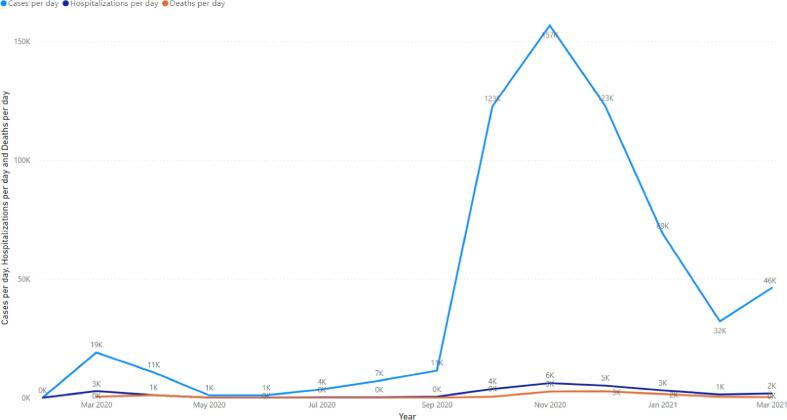
Covid-19 cases, hospitalizations and deaths for Switzerland until March 2021.

**Fig. 5c:**
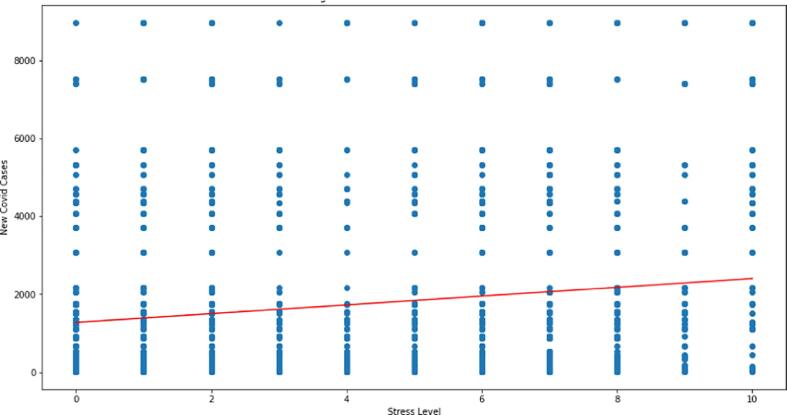
Plot showing correlation between new COVID-19 cases (in 1′000 s) and global stress level (10-points scale).

## Discussion

Between March 2020 and February 2021, 50 surveys were distributed to 127 employees and resulted in 1,877 individual responses and a high response rate of 30% on average. The average global stress level varied significantly by profession, ranging from 2.7 points for administrative staff to 6.9 points for radiation therapy technicians. The average global stress level was highest for in-hospital work with direct patient contact with 4.8 points, whereas stress was similar for in-hospital work without patient contact and home-office with 3.5 and 3.7 points, respectively. Health concerns were highest regarding family and friends with 4.0 points on average compared to concerns about one’s own or patients’ health with an average of 3.1 and 3.6 points, respectively. Changes of response rates and the stress level varied with infection waves.

There is a wealth of literature documenting detrimental health effects on healthcare staff during the extreme conditions of a pandemic, which has been partially updated in light of the COVID-19 pandemic. *Magill et al.* (2020) conducted a rapid review on the mental health of frontline healthcare providers during pandemics. The authors assessed a total of 94 studies and found stress and anxiety to be the most common adverse psychological experiences, which decrease over time during pandemics. This rapid review also evaluated interventions to counter these adverse effects, concluding that more evidence-based measures are needed [4]. *Busch et al.* (2020) conducted a systematic review and *meta*-analysis of the epidemics and pandemics induced psychological burden for healthcare workers over the past two decades. This review of 86 studies, which assessed numerous different psychological and psychosomatic symptom clusters, revealed consistent evidence for significant and detrimental effects on the mental well-being of frontline healthcare workers. The authors close by recommending easy access to support structures for all staff involved in healthcare provision for the entire duration of the COVID-19 pandemic [5]. *Cabarkapa et al.* (2020) provided the most concise, detailed and well-planned rapid systematic review on the psychological impact of COVID-19 and other viral epidemics on frontline healthcare workers. Only studies with an established quantitative or qualitative methodology to assess psychological impact were included, totaling at 52 studies, which comprised various countries and staff during the SARS, Ebola, MERS and COVID-19 outbreaks. This review reported an increased risk for trauma, stress-related disorders, depression, and anxiety. Many examined studies stressed the need for increased offers for psychosocial support, and the need for a more transparent and clear communication strategy throughout viral outbreaks [6].

Since the beginning of the COVID-19 pandemic in the spring 2020, an abundance of online surveys has been conducted examining people’s stress and anxiety levels, vaccination willingness, attitudes towards the national pandemic management and social behavior, among others [13], [14], [15], [16]. Surprisingly, many survey reports did not provide survey response rates, yet those that did, reported survey response rates ranging from 8 to 50% [7], [13]. Our average response rate of 30% appears high considering that this was achieved in a total of 50 repetitive surveys over a duration of one year. A comparatively short survey most likely contributed to this continuously high response rate, together informing staff about the survey results and actions derived from the survey results [7].

In the pre-pandemic radiation oncology practice, almost all professions had regular patient contact. Yet while remote work in the home office setting was partially or fully implemented for some professions such as administrative staff, research staff, medical physicists or senior clinicians during the pandemic, due to the nature of their work as frontline healthcare workers, such arrangements were less likely to be implementable for nurses and RTTs. Consequently, these two professions had significantly higher average global stress levels and health concerns, with RTTs being most affected across all dimensions. In contrast to other international reports and policies, our department did not implement patient waiting times, deferral of radiotherapy treatment or changes of radiotherapy fractionation, and continued treatment of COVID-19 positive patients if their health status allowed so, which was obviously most difficult for RTTs and nurses [17], [18], [19]. This finding is echoed in numerous other studies for various medical sub-specialties. For example, *Morassaei et al.* (2021), in examining stress levels in RTTs in Canada and Norway, found high stress levels during the pandemic and identified similar stressors in both countries [7]. *Vancappel et al.* (2021) in surveying 1,010 healthcare workers at university hospitals in France from across different medical sub-specialties found a high prevalence of post-traumatic and burnout symptoms at the beginning of the COVID-19 pandemic [15].

In this context, it is important to highlight that these findings all originate from countries with largely publicly funded healthcare systems in high-income countries, raising the question whether similar findings would prevail when assessing countries with mostly privately funded or underfunded healthcare systems and/or more pronounced organizational hierarchies. Under such conditions, a lack of easily accessible informal support systems, institutionalized worker support such as labor unions, or even the recognition of the importance of staff wellbeing might influence stress levels and supportive interventions in systems with less favorable environments.

While low global stress level and health concern scores for administrative and research staff may be influenced by the possibility to work from home, it is also important to point out that there does not seem to be a strong correlation between patient contact and employee stress level and health concerns. While clinicians in our department had regular patient contact during in-hospital work, their average global stress levels and health concerns were consistently lower than those of the non-patient facing professions. This finding is both encouraging and concerning at the same time: Encouraging, as it can be interpreted as evidence for empathetic and resilient character traits in the physician profession as well as the ability to immediately integrate new scientific evidence in light of an emerging disease into their day-to-day activities; but also concerning, as amongst physicians it is often expected that one’s own health and wellbeing comes last, as anecdotally exemplified in the self-reflections of a Canadian emergency physician suffering from mental illness [20], which can result in burn-out and depression. No doubt, additional factors are at play, which influence the experience of stress. For example, staff with higher stress levels may have been more likely to fill the survey in the first place to make their voice heard, thereby potentially introducing an upward bias into stress and health concern reporting. Other studies have identified additional levers for healthcare worker stress and provided some first evidence that even within professions global stress levels and health concerns may vary substantially, and also that workplace atmosphere is heavily influenced by such factors as socioeconomic background, gender, ethnicity, age, and career stage [21], [22], [23].

Changes of the average global stress level and health concerns over the 50 surveyed weeks during the first year of the COVID-19 pandemic were graphically seemingly associated with the developments of COVID-19 in Switzerland. After the first wave in the winter 2020, average global stress level and health concerns dropped during the summer months, before rising to the all-time high during the first year of the COVID-19 pandemic at the height of the second wave in the winter 2021. We also observed that during the first and second infection waves, the weekly response rates increased, highlighting the healthcare worker’s heightened alertness, global stress levels and health concerns. The missing statistical evidence for a clear and statistically significant correlation between global stress levels and daily new COVID-19 cases results most likely from the comparatively high global stress levels during the first wave as well as the persistence of high global stress levels after the end of the second wave. Moreover, the effect, which is frequently described in the literature [4], that with every additional month or year a pandemic lasts, employees adapt, leading to decreased stress levels and health concerns, does also not hold for the average global stress level in this study population. However, it is indeed correct that the concerns about health during the second COVID-19 wave did mostly not reach the same levels as during the first COVID-19 wave, though they were higher in November and December 2020.

These findings may become comprehensible when looking at the continuous interventions on a hospital- and departmental-level to order to address the COVID-19 pandemic and healthcare worker wellbeing: Hygienic and organizational adjustments of the USZ Department of Radiation Oncology allowed that all referred patients were treated without waiting times and without adjustment of our standard treatment concepts and fractionation. By March 20th, 2020, protective masks were mandatory for all staff, comprehensive social distancing was implemented, all meetings were shifted in a virtual environment (Microsoft® *Skype for Business*), and employees worked remotely from home whenever possible. In addition, all departmental-level standard operating procedures (SOP) were changed from a horizontal organization (one clinician responsible for one patient in all steps of the treatment chain) to a vertical organization (clinicians responsible for certain workplaces and tasks), alongside with a fully paperless workflow, aiming to minimize “travelling” and contacts within the department; similarly, travelling between all workplaces was kept to a minimum and responsibilities adjusted in all other professions. Simultaneously, anti-stress interventions were designed and implemented in our department. For example, hospital leadership started sharing regular email updates regarding new health authority policies and explaining implications for all hospital staff. This included information on infection rates, hospital capacity and operations as well as effectiveness of personal protective equipment (PPE). Departmental leadership also circulated emails informing about latest developments, evolving new evidence, risk assessments and best practices to be followed. Moreover, during inter-professional departmental leadership meetings, the weekly survey results were analyzed and profession-specific interventions were developed and implemented. For instance, a meditation room was created for all staff and regularly used; voluntary virtual discussions were offered for each professional group; and a communication training for resident physicians was developed, where difficult situations were discussed and reflected upon with a professional coach. A hospital-wide telephone hotline for USZ employees was another resource, which was well received and regularly used by different professional groups, to lower global stress and levels and health concerns.

It is a strength of this study to have surveyed the whole interprofessional landscape of a radiation oncology department during the first year of the COVID-19 pandemic, starting only 20 days after the WHO declared COVID-19 a pandemic. The survey was also designed in a way which made it feasible to answer easily and continuously. It is a shortcoming of this study that no baseline data on global stress level and health concerns was available; a multi-institutional study would have increased statistics power and would have been interesting for external validation of our findings. In addition, as this survey followed an anonymous design, no individual longitudinal analyses were possible, preventing us from looking into psychological coping strategies such as response shifts within different professions. It more generally is a shortcoming of this study that the efficacy of anti-stress interventions was not assessed. Future research efforts should explore the possibility of routine employee surveys, which ideally should address some of all of these limitations.

In conclusion, weekly online surveys for prospective assessment of stress levels and health concerns were successfully conducted during the first year of the COVID-19 pandemic, indicating their feasibility and value to monitor profession and workplace specific stress patterns and to allow for tailored interventions. The physical and mental health of frontline healthcare workers in radiation oncology should remain a top priority for departmental and hospital leadership beyond the COVID-19 pandemic.

## Funding

Sebastian M. Christ received support through the “Young Talents in Clinical Research” Beginner’s Grant from the Swiss Academy of Medical Sciences (SAMW) and the Bangerter-Rhyner Foundation.

*Availability of data and material:* Survey responses are be available upon request.

*Code availability:* Not applicable for this publication.

*Ethics approval:* The Swiss Cantonal Ethics Committee was informed about the study.

*Prior presentation:* The abstract of this manuscript was presented as a poster at the 2021 European Society of Therapeutic Radiology and Oncology (ESTRO) annual meeting.
