# Supplementary material for: Prospective assessment of stress and health concerns of radiation oncology staff during the COVID-19 pandemic
Source: Clin Transl Radiat Oncol. 2022 Jun 8;35:110–7. doi: 10.1016/j.ctro.2022.06.001 (PMC9176183; doi:10.1016/j.ctro.2022.06.001)
Supplement: Supplementary data 1 [file mmc1.docx]

**Supplements 1:** COVID-19 survey questions

(1) Please select your profession from the drop-down menu:

[1] Administrative staff

[2] Clinician

[3] Medical physicist

[4] Nurse

[5] Nursing assistant

[6] Research staff

[7] Radiation therapy technician (RTT)

[8] *Other*

(2) What was the setting of work carried out during the past week?

[1] Onsite with patients

[2] Onsite without patients

[3] Offsite/home office

(3) What is your current overall stress level?

0 [ ] 1 [ ] 2 [ ] 3 [ ] 4 [ ] 5 [ ] 6 [ ] 7 [ ] 8 [ ] 9 [ ] 10 [ ]

*(“0” = no stress; “10” = maximal stress)*

(4) How concerned are you currently about your own health?

0 [ ] 1 [ ] 2 [ ] 3 [ ] 4 [ ] 5 [ ] 6 [ ] 7 [ ] 8 [ ] 9 [ ] 10 [ ]

*(“0” = no stress; “10” = maximal stress)*

(5) How concerned are you currently about the health of your family and friends?

0 [ ] 1 [ ] 2 [ ] 3 [ ] 4 [ ] 5 [ ] 6 [ ] 7 [ ] 8 [ ] 9 [ ] 10 [ ]

*(“0” = no stress; “10” = maximal stress)*

(6) How concerned are you currently about the health of your patients?

0 [ ] 1 [ ] 2 [ ] 3 [ ] 4 [ ] 5 [ ] 6 [ ] 7 [ ] 8 [ ] 9 [ ] 10 [ ]

*(“0” = no stress; “10” = maximal stress)*
